# Genetic and Epigenetic Regulation in Nonalcoholic Fatty Liver Disease (NAFLD)

**DOI:** 10.3390/ijms19030911

**Published:** 2018-03-19

**Authors:** José A Del Campo, Rocío Gallego-Durán, Paloma Gallego, Lourdes Grande

**Affiliations:** 1 Valme University Hospital, Department of Digestive Diseases & CIBERehd, Servicio Andaluz de Salud, University of Sevilla, 41014 Sevilla, Spain; palgalyer@alum.us.es (P.G.); lourdes.grande.sspa@juntadeandalucia.es (L.G.); 2 Instituto de Biomedicina de Sevilla (IBiS) & CIBERehd, 41012 Sevilla, Spain; rociogallegoduran@gmail.com

**Keywords:** NAFLD, genetics, epigenetics, miRNAs, SIRT1, *PNPLA3*

## Abstract

Genetics and epigenetics play a key role in the development of several diseases, including nonalcoholic fatty liver disease (NAFLD). Family studies demonstrate that first degree relatives of patients with NAFLD are at a much higher risk of the disease than the general population. The development of the Genome Wide Association Study (GWAS) technology has allowed the identification of numerous genetic polymorphisms involved in the evolution of diseases (e.g., *PNPLA3*, *MBOAT7*). On the other hand, epigenetic changes interact with inherited risk factors to determine an individual’s susceptibility to NAFLD. Modifications of the histones amino-terminal ends are key factors in the maintenance of chromatin structure and gene expression (cAMP-responsive element binding protein H (CREBH) or SIRT1). Activation of SIRT1 showed potential against the physiological mechanisms related to NAFLD. Abnormal DNA methylation represents a starting point for cancer development in NAFLD patients. Besides, the evaluation of circulating miRNA profiles represents a promising approach to assess and non-invasively monitor liver disease severity. To date, there is no approved pharmacologic therapy for NAFLD and the current treatment remains weight loss with lifestyle modification and exercise. In this review, the status of research into relevant genetic and epigenetic modifiers of NAFLD progression will be discussed.

## 1. Introduction

In 1980, a number of patients who had histopathological changes indistinguishable from alcoholic liver disease were described for the first time, but in whom alcohol intake was nil or non-significant [1]. Nonalcoholic fatty liver disease (NAFLD) comprises a progressive spectrum of diseases ranging from hepatic steatosis-accumulation of fat in hepatocytes from an initially benign character to nonalcoholic steatohepatitis, characterized by inflammatory infiltrate and hepatocyte damage (which includes ballooning and cell death) together with deposition of collagen and fibrosis progression, although simple steatosis may promote fibrosis progression [2]. This disease is diagnosed in the absence of significant alcohol consumption, presence of other hereditary diseases, consumption of drugs that can induce steatosis and viral infections (hepatitis B and C virus, human immunodeficiency virus (HIV)) [3].

Simple steatosis and steatohepatitis show different natural histories and consequences. This pathology has been associated with an increased mortality rate, 5–10 times higher than in the general population [4]. After the establishment of steatohepatitis, up to 30% of patients can develop cirrhosis and hepatocellular carcinoma (HCC) in 10 years, although HCC may typically also develop in non-fibrotic livers and simple steatosis may also have a progressive course [4,5]. In addition, steatohepatitis is considered the greatest predictor of fibrosis progression and has been described as a key pathogenic process related to systemic disorders, especially cardiovascular risk [6,7,8,9].

NAFLD is currently considered the most common liver disease in developed countries [10]. Its global prevalence in the general population is variable; in the Eastern countries it is 20–30% and in Asia 5–18% [11]. The reason for this variability is not clear yet, but presumably genetic and epigenetic factors could play a major role. The incidence of NAFLD is two new cases per 100 people per year, and up to 50% of the general population is expected to be affected by 2030. Despite being a very common condition, NAFLD tends to be under-diagnosed.

Its development is closely related to the characteristics of the metabolic syndrome, such as central obesity, insulin resistance, type 2 diabetes mellitus, arterial hypertension and dyslipidemia [12]. In discussing the biological mechanisms underpinning the relationship between NAFLD and chronic vascular complications of diabetes mellitus, there is emerging evidence that suggests a link between dysbiosis, intestinal barrier dysfunction, mediators of the gut microbiota and cardiovascular diseases [13]. Hepatic fat accumulation generates multiple signals that alter lipid and glucose metabolism, leading to the presence of intracellular fat vacuoles, lack of the ability to perform the mitochondrial beta-oxidation process, generation of oxidative stress, development of pro-inflammatory mechanisms and, finally, hepatocellular apoptosis [14,15,16].

Liver biopsy is the current reference method for the diagnosis and staging of both fibrosis and steatohepatitis. It is usually recommended to patients with elevated transaminases with no affiliated origins and for which other risk factors have been excluded. However, it is an invasive, expensive and time-consuming method, presenting inherent risks, since it is associated with increased morbidity, which in some cases can lead to complications such as bleeding and even death [17]. Among pathologists, the most common method for diagnosis is the NAS score (NAFLD Activity Score) [17] which provides a score based on the degree of steatosis (0–3), lobular inflammation (0–3) and ballooning degeneration (0–2). A score >5 would be confirmatory of steatohepatitis, whereas if it is inferior to 3, this injury would be unlikely. In addition, taking into account that common treatments for this pathology are still ineffective, the first line of action for this type of patients would be weight loss, which would be achieved with a decrease in caloric intake and an increase in physical activity. Lifestyle intervention can be effective when treating non-alcoholic fatty liver diseases (NAFLD) patients and clinical evidence strongly supports the role of lifestyle modification as a primary therapy for the management of NAFLD and nonalcoholic steatohepatitis (NASH) [18]. 

To establish proper diagnosis, prognosis and appropriate therapeutic management, it would be essential to differentiate between steatohepatitis and simple steatosis, taking into account the systemic complications of this disease. Besides, diagnosing fibrosis represents a key role, since its presence is a major determinant of the natural course of hepatic and extra-hepatic disease.

## 2. Genetics in NAFLD

There is a considerable inter-individual variation in terms of severity and risk of morbidity and mortality associated with NAFLD. The pathogenesis of NAFLD is thought to be a multifactorial and complicated disease associated with lifestyle habits, nutritional factors and genetics. However, the pathogenesis and underlying mechanism in the development of NAFLD caused by genetics remains unclear. The function of gene polymorphisms, which implicate Insulin resistance (IR), fatty acid metabolism, oxidative stress and hepatofibrogenesis, are reflected in every part of the pathogenesis of NAFLD [19]. 

The development of the Genome Wide Association Study (GWAS) technology has allowed the identification of numerous genetic polymorphisms involved in the evolution of diseases, since they can alter the stages of development, susceptibility to disease, rate of progression and efficacy of the treatment. In 2008, the first GWAS was published in NAFLD [20] patients and 9229 single nucleotide polymorphisms (SNPs) were analyzed in a population of varied ethnicity in order to search for genetic variants associated with the disease. This study detected that SNP rs738409 of the gene-like phospholipase domain-containing protein 3 (*PNPLA3*) was strongly associated with accumulation of fat in the hepatocyte in a cohort of more than 2000 ethnically diverse patients. This association remained significant even though it was adjusted for body mass index (BMI), diabetes mellitus, alcohol intake and ethnicity. Later, other approaches of this type have been carried out, identifying new candidate genes, but in which *PNPLA3* has always remained significantly associated to disease development [21]. Numerous studies have validated this relationship, and new findings have been made, such as its relationship to alananine transaminase (ALT) serum levels and the full spectrum of NAFLD, including simple steatosis, steatohepatitis, cirrhosis and hepatocellular carcinoma, as well as systemic disorders more frequent in these patients, such as cardiovascular risk. Numerous studies have validated this relationship [22,23], and new findings have been made, such as its relationship to ALT [24] serum levels and the full spectrum of NAFLD, including simple steatosis, steatohepatitis, cirrhosis and hepatocellular carcinoma [25,26], as well as systemic disorders more frequent in these patients, such as cardiovascular risk.

In humans, *PNPLA3* is located on the long arm of chromosome 22. The specific SNP rs738409 (rs738409 C>G) has the peculiarity that it encodes an amino acid substitution from isoleucine to methionine at position 148; I148M). *PNPLA3* encodes a triacylglycerol lipase, also called adiponutrin, which is involved in the hydrolysis of triacylglycerol in the adipocytes. Such a hydrolase activity may not be present in those patients presenting the I148M variant [27], since this amino acid substitution near the catalytic domain reduces its enzymatic activity and favors the development of steatosis [28], which in other words would be considered a loss of function. This would have an impact similar to other factors, such as environmental stress, obesity or alcohol consumption, affecting the predisposition for the disease to progress. This protein is expressed in the endoplasmic reticulum, hepatocyte lipid membranes and adipose tissue [29]. It has been also demonstrated that adiponutrin circulates in human plasma, and that its concentration oscillates between 1.25 and 4 nM, posing as a possible biomarker of the disease [30].

Basuray et al. [31] have generated a line of knock-in mice introducing a methionine codon at position 148 of the Pnpla3 gene in female C57BL/6J mice. This study provides solid evidence to affirm that the presence of this variant favors the development of NAFLD. These mice show normal levels of fat in the liver while maintained on a standard diet. When a sucrose-rich diet is applied, intrahepatocyte fat levels were increased 2–3 times more compared to controls, without detecting changes in glucose homeostasis. In addition, these mice showed an increase of up to 40-fold in the levels of catalytically inactive adiponutrin in the lipid droplets, without overexpression of *PNPLA3* mRNA in the liver.

In recent years, the deleterious effect of the G allele of this *PNPLA3* SNP has been described. This has been associated with an additive effect in the development of a broad spectrum of liver diseases, such as alcoholic cirrhosis [32], viral hepatitis [33], and even hepatocarcinoma [25]. The carriers of the G allele are 2.26 times more likely to develop liver cancer, with GG homozygotes up to five times more at risk. In addition, it has been described that although carriers of the GG genotype have an increased risk of developing NAFLD, they are also more susceptible to the beneficial effects that an intervention provides at the level of lifestyle [34].

Recently, it has been shown that the *MBOAT7* locus is associated with increased hepatic fat content in two cohorts and with the entire spectrum of histological liver damage related to NAFLD [35]. This association is mediated by lower hepatic protein expression of Membrane Bound O-Acyltransferase Domain Containing 7 (MBOAT7) resulting in changes in the hepatic phosphatidylinositol acyl-chain remodeling. Moreover, it has been demonstrated that the MBOAT7 rs641738 T allele is associated with reduced MBOAT7 expression and may predispose to HCC in patients without cirrhosis, suggesting it should be evaluated in future prospective studies aimed at stratifying NAFLD-HCC risk [36].

Besides *PNPLA3* and *MBOAT*, other genetic variants have been described to promote NAFLD progression. These variants have been summarized in Table 1. In this sense, transmembrane 6 superfamily member 2 (*TM6SF2*) is involved in the enrichment of triglycerides to apolipoprotein B100 in the pathway of very low-density lipoprotein secretion from the hepatocytes [37] . The rs58542926 C>T polymorphism in this gene results in a loss-of-function, inducing higher liver triglyceride content and lower circulating lipoproteins. In experimental models, silencing of TM6SF2 reduces secretion of very-low-density lipoproteins (VLDLs) and causes a predisposition to retention of triglycerides (TGs) in hepatic lipid droplets and fatty liver [37,38]. Dongiovanni et al. [39] have shown that TM6SF2 E167K variation is associated with NASH, hepatocellular ballooning, and necroinflammation in humans. Moreover, a robust association between this variant and histological severity of steatosis and fibrosis has been shown.

Variation in the glucokinase regulator (GCKR) gene locus has been also associated with NAFLD [40]. GCKR regulates de novo lipogenesis by controlling the influx of glucose in hepatocytes. Loss-of-function GCKR mutation (rs1260326) encoding the P446L protein variant, leads to decreased circulating fasting glucose and insulin levels and increased hepatic fat accumulation by blocking fatty acid oxidation [39]. 

The association between the IFN-λ3/IFN-λ4 locus, hepatic inflammation and fibrosis has recently been confirmed in a large multi-center cohort of patients with NAFLD, in whom the impact of this variant was larger in non-obese than obese individuals [41,42]

## 3. Epigenetics in NAFLD

Historically, the term epigenetics was introduced by Conrad Waddington in 1940. It was defined as the branch of biology that studies the causal interactions between genes and their products that originate the phenotype. Nowadays, epigenetic phenomena are defined as changes in hereditary gene expression through mitosis and/or meiosis, caused by an adaptive mechanism that is not related to alterations of the primary DNA sequence [43]. These mechanisms are considered reversible, since they are modulated by environmental stimuli, and their imbalance can lead to the development of a wide spectrum of disorders of varying severity.

### 3.1. Histone Modifications

Modifications of the histones amino-terminal ends are key factors in the maintenance of chromatin structure and gene expression. It has been described that aberrant histone modifications promote the development of insulin resistance and, consequently, NAFLD [44]. Among the main modifications are acetylations, related to the activation of gene transcription and catalyzed by histone acetyltransferases (HAT), and deacetylations, involved in repression of genes and catalyzed by histone deacetylases (HDAC). Up to date, the greatest findings have been described in mice. The imbalance between both enzymes seems to influence the phenotypic gene expression in NAFLD, resulting in liver damage [45].

Zhang et al. [46] have defined CREBH to be a stress-inducible transcriptional activator that is critically involved in inflammation and metabolism. Metabolic stress and acute liver injuries can induce CREBH cleavage, mediated by the site 1 protease (SP1) and site 2 protease (SP2), the same enzymes that process sterol regulatory element binding proteins upon the demands of lipid or sterol biosynthesis [47]. The cleaved N-terminal fragment of CREBH enters into the nucleus and acts as a potent transcription factor that activates the expression of genes involved in the hepatic acute-phase response, gluconeogenesis, lipogenesis, fatty acids (FA) oxidation, and lipolysis [48]. Modulation of CREBH acetylation can significantly affect CREBH transcriptional activity and lead to the altered lipid homeostasis associated with hepatic steatosis and hyperlipidemia [49]. The deacetylase sirtuin-1 (SIRT1) is known to mediate the nutritional and hormonal modulation of hepatic energy metabolism through the deacetylation of metabolic regulators. The activation of SIRT1 shows potential against the physiological mechanisms related to NAFLD, and its plasma levels have been found to be increased in obese patients with NAFLD [50]. The beneficial effect of SIRT1 activation observed in experimental models of NAFLD has been linked to modulation of peroxisome proliferator-activated receptor α (PPARα) activity and fatty acid oxidation [51] more than to chromatin remodeling. Therefore, SIRT1 could play a dual role, on the one hand as a potential therapeutic target and also as a non-invasive biomarker of NAFLD.

### 3.2. DNA Methylation

DNA methylation is the addition of a methyl group to a cytosine with guanine as the next nucleotide (CpG islands). DNA methylation plays a central role in the regulation of gene expression, representing a level of epigenetic regulation commonly associated with transcriptional repression and chromatin accessibility. Aberrant patterns of DNA methylation affect cellular homeostasis, such as hypermethylation, associated with gene repression, and hypomethylation, related to gene activation. Many methylations take place in the liver, so that hepatic steatosis is commonly seen from the point of view of the deregulation of carbon metabolism, being related to folate deficiency [52]. In mice, the onset of steatosis is accompanied by alterations in the expression of DNA methyltransferases (DNMTs) in the liver [53]. In humans, hepatic DNMT levels have been found to be increased in patients with steatohepatitis versus those with simple steatosis, and in addition to being significantly associated with the NAS Score [54].

Abnormal DNA methylation seems to be the starting point for carcinogenesis, especially in NAFLD-related hepatocellular carcinoma [55]. Metabolites derived from the metabolic syndrome, such as insulin, glucose or lipids, could disrupt gene regulation at the epigenetic level, leading the body to a pro-inflammatory state disrupting metabolic pathways [56]. In addition, DNA methylation can be remodeled by transcriptional factors. They have been evaluated after bariatric surgery and the massive weight loss that it entails, suggesting that changes in methylation associated with NAFLD may be partially reversible [57,58]. Differential methylation may contribute to significant differences in gene expression; 69,247 CpG sites with a different methylation pattern (78% hypomethylated, 24% hypermethylated) have been described in liver tissue of 100 patients with different stages of disease [59,60]. It has been shown in humans diagnosed with NAFLD that PPARs are hyper-methylated and fibrogenic TGF-β1 and PDGF-α are hypo-methylated in patients with progressive disease [61]. Moreover, preliminary evidence suggests that circulating cell-free DNA reflects PPARγ methylation status in the liver, representing a potential non-invasive biomarker of liver damage [62].

### 3.3. MicroRNAs

MicroRNAs are a class of single chain small non-coding RNA molecules that act as regulators of gene expression and participate in translation of proteins. They can interfere with every aspect of cellular activity, such as differentiation and development, proliferation, metabolism, apoptosis and carcinogenesis [63]. These molecules are receiving increasing attention since they are commonly deregulated in pathological situations, and are currently the most intensely studied epigenetic factors in NAFLD.

The targets of microRNA can be multiple genes (multiplicity), or multiple microRNAs can target a single gene (cooperativity). In addition, considering their potential in carcinogenesis, microRNAs can be categorized as oncogenes or onco-microRNAs or as tumor suppressor genes [64]. miRNAs stability has been demonstrated in serum, plasma, saliva and urine. The microRNAs circulate protected from the degradation of RNases contained in body fluids, and are currently being widely studied for the non-invasive diagnosis of a series of liver diseases, including NAFLD [65]. These findings suggest that aberrant expression of miRNAs may have mechanistic significance in NASH-associated liver carcinogenesis and may serve as an indicator for the development of NASH-derived HCC. An overview of the role of miRNAs in NAFLD progression in humans is given in Table 2.

Actually, the greatest importance of microphones in NAFLD lies in its potential to discriminate steatohepatitis and the diagnosis of hepatocarcinoma. In this sense, miR-122, the most expressed microRNA in human liver, is inhibited in steatohepatitis [72], acting as a suppressor of tumors in the liver. Silencing of miR-122 is an early event during hepatocarcinogenesis from NASH, and miR-122 could be a novel molecular marker for evaluating the risk of HCC in patients with NASH [73]. It has been also proposed as a potential therapeutic target in the treatment of hypercholesterolemia and other dyslipidemias [66].

Besides miR-122, others miRNAs have been identified to be involved in NAFLD progression: Pirola et al. identified significant fold differences in serum levels of miR-192 (4.4-fold change in NASH vs. controls) [67]; Cermelli et al. identified increased serum levels of miR-34a and miR-16 in NAFLD patients compared to controls [68]; Yamada et al. identified in a cohort of NAFLD-diagnosed patients increased serum abundance of miR-21, miR-34a and miR-451 relative to controls [69]; Tan et al. identified miR-1290, miR-27b-3p and miR-192-5p as a panel of high diagnostic accuracy for NAFLD [65]; Guo et al. reported three miRNAs (miR-301a-3p, miR-34a-5p and miR-375) as potential biomarkers to access the severity of NAFLD [74]. Recently, it has been proposed that maintaining miR-155 expression in inflammatory cells might be a potential strategy to modulate liver injury [71]. 

There are still some barriers to the therapeutic use of miRNAs, due to their degradation by endogenous RNases. In addition, they affect several routes in different organs, so one still has to treat them with caution to avoid possible unwanted side effects. However, by characterizing the dysregulated miRNAs in the circulation, we might be able to identify key signaling pathways involved in the pathogenesis of the disease and also identify individuals at risk through miRNA profiling.

## 4. Concluding Remarks

Genetics and epigenetics play a key role in the development of several diseases, including NAFLD. New genetic determinants of NAFLD will continue to appear in the coming years, where future strategies should increase the number of individuals included in GWAS. Moreover, epigenetic changes interact with inherited risk factors to determine an individual’s susceptibility to NAFLD. To date, there is no approved pharmacologic therapy for NAFLD and the current treatment remains lifestyle modification with dieting and exercise aiming at weight loss. Therapeutic approaches involve potential modulation of the activity of enzymes responsible for DNA and protein epigenetic modifications. On the other hand, gene expression regulation is also provided by non-coding RNAs (miRNAs). The evaluation of circulating miRNA profiles represents a promising approach to assess and non-invasively monitor liver disease severity, although this issue still requires independent validations.

## Figures and Tables

**Table 1:** Genetic variants (SNPs) associated to NAFLD progression.

Gene	Function	Phenotype	Variant
*PNPLA3*	Lipid droplets	↑ NAFLD, NASH, fibrosis, HCC	rs738409 C>G
*MBOAT7*	Phospholipid metabolism	↑ NAFLD-HCC risk	rs641738 C>T
*APOB*	VLDL secretion	↑ NAFLD, NASH, fibrosis, HCC	Several
*TM6SF2*	VLDL secretion	↑ NAFLD, NASH, fibrosis	rs58542926 C>T
*GCKR*	De novo lipogenesis regulation	↑ NAFLD, NASH, fibrosis	rs780094 A>G
*KLF6*	De novo lipogenesis regulation; fibrogenesis	↓ fibrosis	rs3750861 G>A
*IL28B*	Innate immunity, alternative IFNL3/4 transcription	↓ fibrosis	rs12979860 C>T
*SOD2*	Mitochondrial antioxidant	↑ fibrosis	rs4880 C>T

*APOB*, Apolipoprotein B; *KLF6*, Krueppel-like factor 6; *IL28B*, interleukin 28B; *SOD2*, Superoxide dismutase 2; VLDL: very low-density lipoproteins. ↑—increased, ↓—decreased.

**Table 2:** Association of specific miRNAs with NAFLD development and progression in humans.

miRNA	Pathway	Reference
miR-122	Lipid metabolism, carcinogenesis	[65]
miR-192	NAFLD progression	[66]
miR-34a, miR-16	Lipid metabolism	[67]
miR-21, miR-34a	Lipid metabolism	[68]
miR-1290, miR-27b-3p and miR-192-5p	Several (panel)	[64]
miR-301a-3p, miR-34a-5p and miR-375	Several (biomarkers)	[69]
miR-182	Fibrogenesis	[70]
miR-155	Inflammatory pathway and liver injury	[71]
